# A Case of Recurrent Renal Aluminum Hydroxide Stone

**DOI:** 10.1155/2014/212314

**Published:** 2014-06-09

**Authors:** Basri Cakıroglu, Akif Nuri Dogan, Tuncay Tas, Ramazan Gozukucuk, Bekir Sami Uyanik

**Affiliations:** ^1^Hisar Intercontinental Hospital, Department of Urology, Umraniye, 34768 Istanbul, Turkey; ^2^Hisar Intercontinental Hospital, Department of Internal Medicine, Umraniye, 34768 Istanbul, Turkey; ^3^Taksim Training and Research Hospital, Department of Urology, Taksim, 34433 Istanbul, Turkey; ^4^Hisar Intercontinental Hospital, Department of Infectious Diseases and Clinical Microbiology, Umraniye, 34768 Istanbul, Turkey; ^5^Hisar Intercontinental Hospital, Department of Clinical Biochemistry, Umraniye, 34768 Istanbul, Turkey

## Abstract

Renal stone disease is characterized by the differences depending on the age, gender, and the geographic location of the patients. Seventy-five percent of the renal stone components is the calcium (Ca). The most common type of the stones is the Ca oxalate stones, while Ca phosphate, uric acid, struvite, and sistine stones are more rarely reported. Other than these types, triamterene, adenosine, silica, indinavir, and ephedrine stones are also reported in the literature as case reports. However, to the best of our knowledge, aluminum hydroxide stones was not reported reported before. Herein we will report a 38-years-old woman with the history of recurrent renal colic disease whose renal stone was determined as aluminum hydroxide stone in type. Aluminum mineral may be considered in the formation of kidney stones as it is widely used in the field of healthcare and cosmetics.

## 1. Background

The estimated lifetime prevalence of kidney stone disease is 1–15%. The probability of having a urinary stone differs according to age, gender, race, and geographic location of the patients. It has been determined that men are affected by stone disease two to three times more commonly than women in adulthood [1].

The most common type of the stones is the calcium oxalate CaOx type which makes up the 60% of all. It is followed by mixed calcium oxalate and hydroxyapatite stones establishing 20% of renal calculi. Calcium, being the major component of approximately 75% of stones, is the most common constituent of urinary calculi. The other reported stones are uric acid and struvite (magnesium ammonium phosphate) stones occurring in 10% of cases and brushite stones with an incidence of 2% in all stones and rarely reported (1%) cysteine stones. On the other hand, some stones associated with medications and their by-products are also reported including triamterene, adenosine, silica, indinavir, and ephedrine [2].

For a long time, essential and toxic trace elements have been suggested to play a role in urinary stone formation. In this topic, it was also recommended to accomplish studies on some trace elements including aluminum or lead [3].

Aluminum is the normal component of raw water and its concentrations are determined to be high in surface waters while it is usually low in amount in ground waters [4]. Aluminum toxicity is an uncommon condition and almost always reported in iatrogenic cases including patients receiving total parenteral nutrition and dialysis patients using aluminum-containing phosphate binders, antacids, and dialysates [5].

Herein we will report a patient with urinary stone containing high aluminum hydroxide component, which is not reported before in the literature.

## 2. Case Report

**Figure 1:**
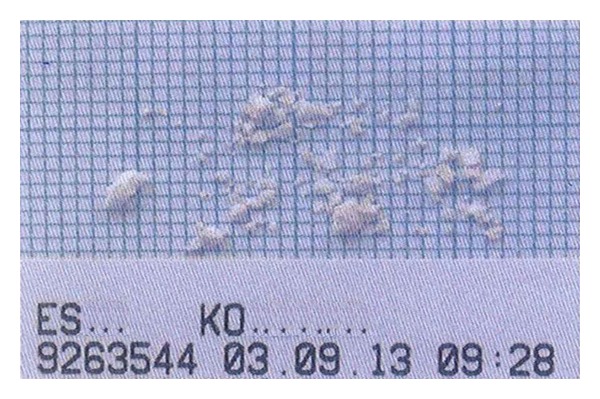
Four of stones broken by hand.

**Figure 2:**
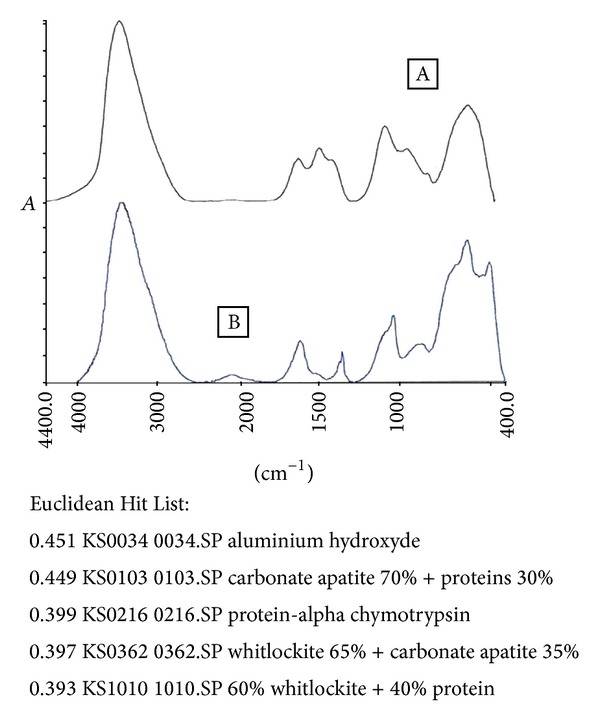
The analysis of stones (Fourier transform infrared spectroscopy).

Thirty-eight-year-old woman with 162 cm height and 76 kg weight was admitted to our outpatient clinic with the complaint of renal colic. She had a history of renal stone diseases for 6 times in the last 6 months with 20–40 days of intermittences. Her stones were white in color and they were easily dispersible with a diameter of 3–5 mm approximately (Figure 1). She had collected her stones and Fourier transform infrared spectroscopy (FTIR) was performed to the stones for the analysis (Figure 2). The results of the analysis revealed that 45.1% of the stones were aluminum hydroxide and 44.9% were carbonate apatite and protein. Metabolic investigations were made both in serum and 24-hour urine in order to determine the etiology of stone (Table 1). Serum aluminum was determined as 32 *μ*g/L (1–14) and calcium was 9.87 mg/dL (8.8–10.2), while urinary calcium excretion was 0.38 g/day (0.1–0.3). In her history it was determined that she was using a roll-on deodorant that was containing aluminum. After 2-month period of dismissing this deodorant, her serum aluminum level was 12 *μ*g/L (1–14) and her urinary aluminum excretion was in normal limits (3.67 *μ*g/day, reference range: <50).

She was evaluated with urinary ultrasound and tomography for the presence of stone disease and they were all normal. In approximately 5 months of follow-up, she did not have any renal colic attacks.

## 3. Discussion

To the best of our knowledge, any data about the aluminum hydroxide stones was not present in the literature among urinary stones up to date. Nowadays, the role of essential and trace elements is being discussed in formation of stones. Though in some studies these trace elements were reported to be present in trace amounts in stone mass, these elements were thought to be not present in high concentrations in the structure of stone [3, 6–8].

Aluminum toxicity is a rare disorder that typically occurs iatrogenically in patients receiving total parenteral nutrition and in dialysis patients taking aluminum-containing phosphate binders, antacids, and dialysates [5]. Aluminum-containing phosphate binders have been used for hyperphosphatemia treatment in dialysis patients for a long time. A cautious follow-up is suggested for dialysis patients using aluminum containing phosphate binders for the increased risk of Al-Mg-urate stone formation.

Inhibitory crystallization deficits and supersaturated levels of different salts, promoters, and inhibitors of crystallization have been determined to play a major role in stone disease formation. In this aspect, the main natural inhibitors of calculi formation are known as citrate, pyrophosphate, nephrocalcin, glycosaminoglycans, magnesium, and zinc [9].

The role of trace elements in urinary stone formation is still unknown and controversial. Durak et al. have studied the distribution of five metals (Iron, copper, cadmium, zinc, and magnesium) in 47 stones and hair of patients and found significant differences among the element levels in stones, patient hair, and control hair [6]. Levinson et al. suggested that some trace elements (such as aluminum and lead) play a role in stone formation and studies about this topic are warranted [3]. In another in vitro study, urinary aluminum was determined to affect the enlargement of calcium oxalate crystals. The writers concluded that though trace elements in calculi did not directly affect the formation of CaOx and calcium phosphate (CaP) stones in normal conditions, they resulted in urinary stone formation with metabolic processes [8]. Previously, in some studies the stones were analyzed completely and in different stone types, the concentrations of trace elements were determined to be different [4–6]. In some studies, the concentrations of some heavy metals (lead, cadmium, nickel, and aluminum) were found to be higher in nuclear region compared with the crust [7].

Aluminum hydroxide was used for many purposes but currently it is often used in medical antiacid treatment and in cosmetic products as preservative. In our case, aluminum stone was presented in mixed type. There was no history of anti-acid or work-exposure to aluminum in our patient. However she was using a cosmetic material containing aluminum and dismissing this product decreased serum aluminum levels and stone recurrences. In 6-month period after dismissing this cosmetic product, any complaints of renal colic or stone disease were not reported.

## 4. Conclusion

In the literature there is no data about the association of increased renal stone disease risk with medically used aluminum hydroxide in patients other than hemodialysis patients.

Aluminum mineral may be considered in the formation of kidney stones as it is widely used in the field of healthcare and cosmetics. This issue should be discussed and should be clarified by further investigations.

## Figures and Tables

**Table 1:** Metabolic investigations in serum and 24-hour urinary excretion.

	Serum		Urine (24 Hours) Volume: 1700 mL	
	Result	Reference range	Result	Reference range
Density	—	—	1020	1010–1025
pH	—	—	6.5	4.5–7.5
Creatinine	0.55 mg/dL	0.51–1.05	1.31 g/day	0.6–1.6
Aluminum	32 *μ*g/L	1–14	3.67 *μ*g/day	<50
Oxalate	—	—	33 mg/day	<45
Citrate	—	—	1132 mg/day	252–1164
Calcium	9.87 mg/dL	8.8–10.2	0.38 g/day	0.1–0.3
Phosphorus	3.32 mg/dL	2.7–4.5	0.65 g/day	0.40–1.3
Magnesium	1.9 mg/dL	1.58–2.55	0.080 g/day	0.060–2
Sodium	136 mEq/L	135–145	160 mEq/day	40–220
Potassium	3.6 mEq/L	3.5–5.5	56 mEq/day	25–125
Parathormone	57 pg/mL	15–68	—	—
Alkaline phosphatase	100 IU/L	35–104	—	—
Gamma glutamyl transferase	30 IU/L	7–40	—	—
